# Gastric and colonic erosion caused by laparoscopic gastric band: a case report

**DOI:** 10.1259/bjrcr.20160135

**Published:** 2017-03-10

**Authors:** Luis E Gonzalez, Rajendra P Kedar

**Affiliations:** Department of Radiology, Tampa General Hospital, University of South Florida College of Medicine, Tampa, FL, USA

## Abstract

Laparoscopic adjustable gastric banding is commonly used to treat obesity. It rarely results in complications, one of which is gastrointestinal erosion. Simultaneous erosion of the stomach and colon is a rare finding that has been documented in only a few case reports. We present a 62-year-old female with abdominal pain, nausea, and hematochezia. She was found to have simultaneous gastric and colonic erosion identified on CT scan. Imaging findings were confirmed and device was removed during surgery.

## Introduction

According to the Centers for Disease Control and Prevention, over one-third of Americans are obese.^1^ Given the increasing prevalence of obesity and limited long-term success with dieting and exercise, many patients have sought various types of bariatric procedures for treatment. Laparoscopic adjustable gastric banding (LAGB) uses a restrictive mechanism to reduce the stomach’s capacity and subsequently limit caloric intake in obese patients. In 2015, LAGB comprised 5.7% of all bariatric surgery cases.^2^ LAGB has several advantages including low mortality rate, adjustability of the outlet to allow flexibility with caloric needs, reversibility, less surgical complications and avoidance of staple lines and prosthetic mesh used in other bariatric techniques. However, LAGB is rarely accompanied by complications that may lead to serious consequences for the patient. In this case, we will discuss a rare complication: simultaneous gastric and colonic erosion after LAGB. To our knowledge, only three similar cases have been previously described in the literature.

## Case presentation

A 62-year-old lady with past medical history of lupus anticoagulant on warfarin, and grade II obesity (body mass index of 38.5) status post laparoscopic gastric banding in 2003, presented with a 3-day history of bright red blood with clots per rectum. She presented with left-lower quadrant abdominal pain, nausea, emesis, lightheadedness, weakness and exertional dyspnea. On evaluation in the emergency department, patient was afebrile and vital signs were within normal limits. Physical examination was unremarkable. Haemoglobin was 9.4 and INR was 2.1 on admission.

## Investigations/imaging findings

**Figure 1.**
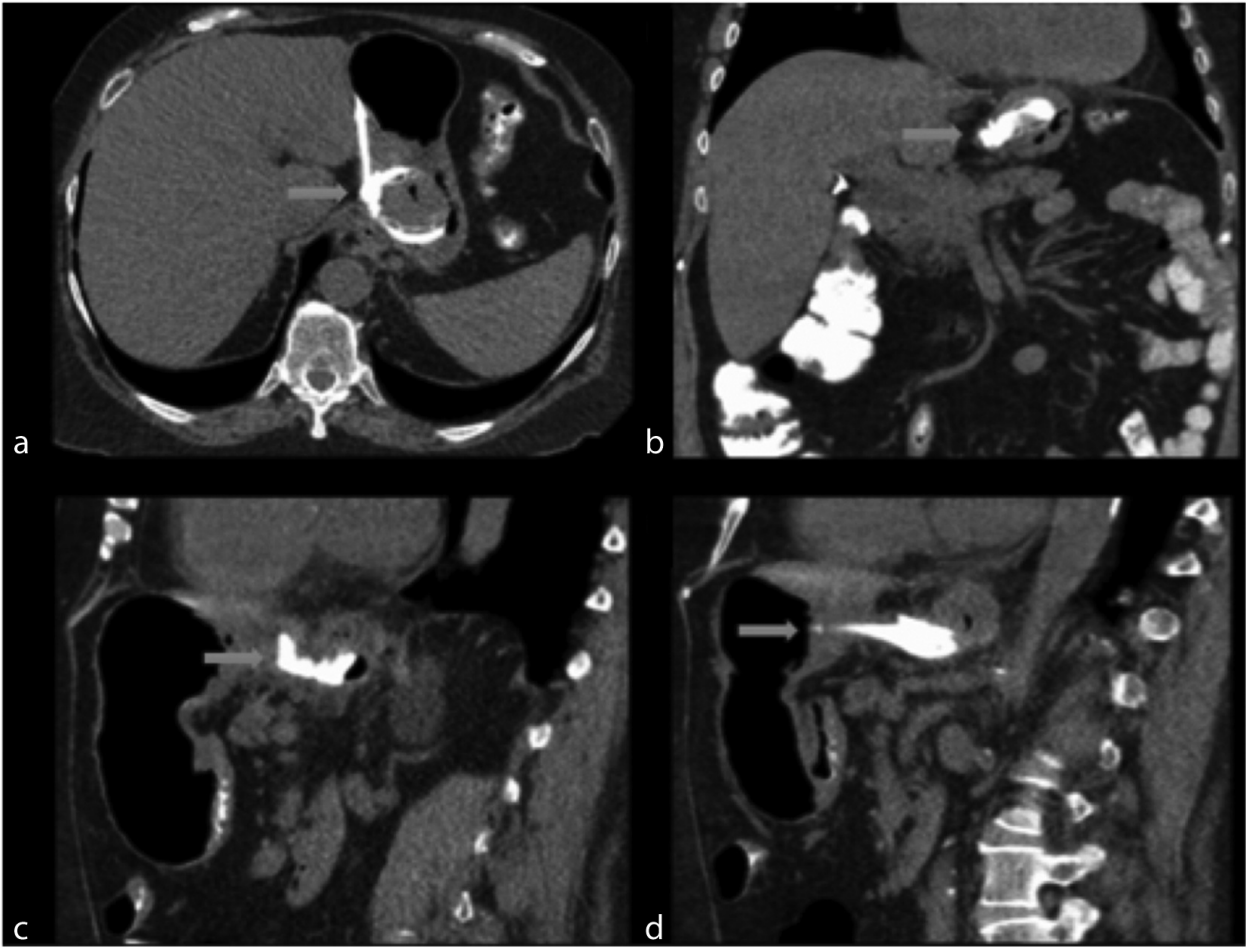
(a,b) Axial and coronal images, respectively, show gastric band erosion. These images illustrate the gastric band (blue arrow) eroded within the gastric lumen in almost its entirety and surrounding inflammatory changes. (c,d) Sagittal images show similar finding.

**Figure 2.**
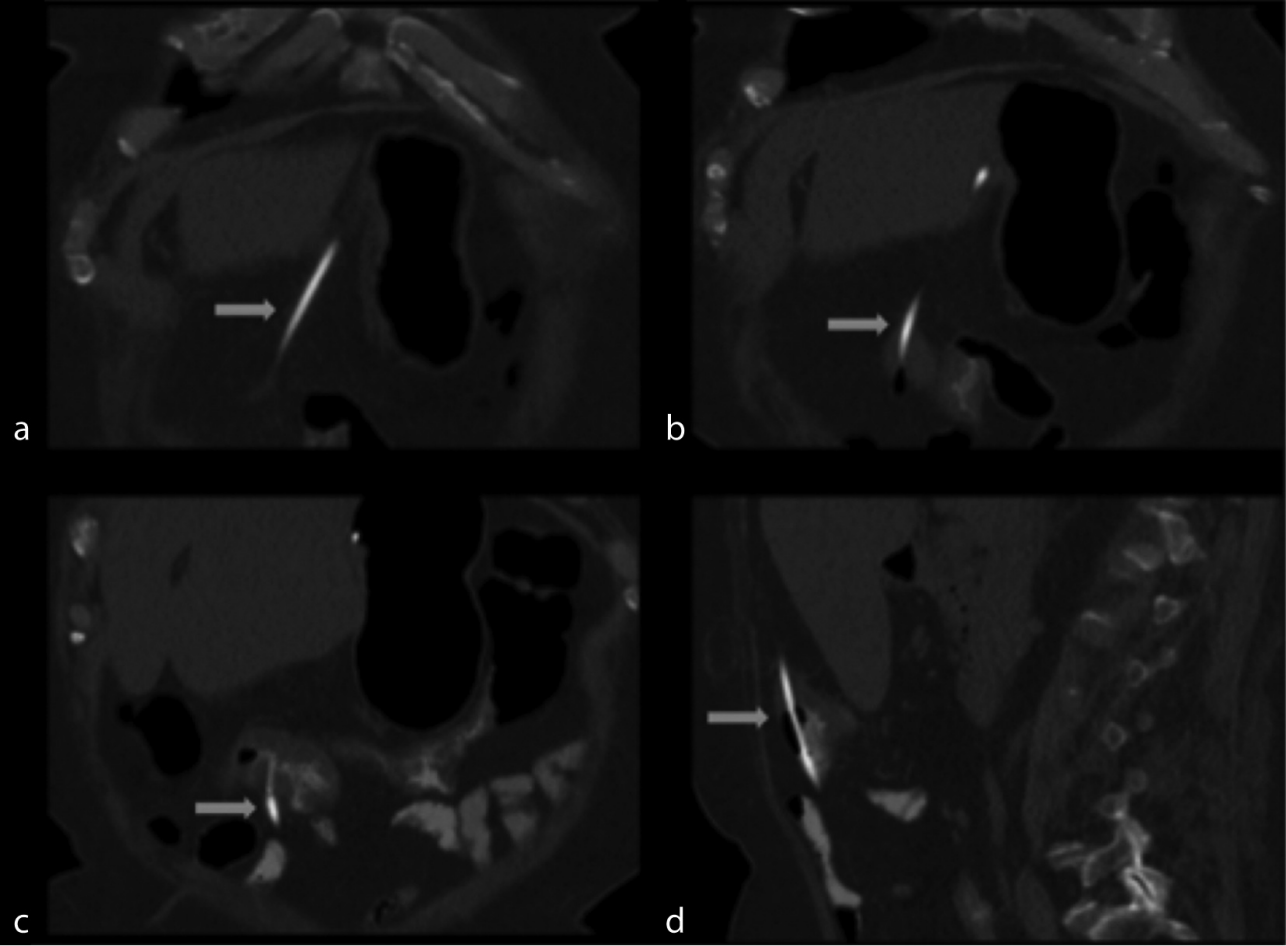
(a–c) Coronal images show the distal part of the connecting tube (blue arrow) extending from the stomach and eroding into the transverse colon with surrounding inflammatory changes. (d) Sagittal image shows similar finding.

Owing to bleeding per rectum, colonoscopy was performed which showed diverticulosis and a foreign body eroding through the transverse colon. CT scan of the abdomen with oral contrast was ordered by the referring physician. It should be noted that the use of positive oral contrast agents is not recommended because it can obscure mucosal enhancement and intraluminal haemorrhage. Ideally, the study should be ordered with and without IV contrast medium. The CT scan showed erosion of the lateral portion of the laparoscopic gastric band into the wall of the stomach (Figure 1). There was also partial excision of the distal portion of the laparoscopic gastric band tubing with erosion of a free distal tip into the transverse colon (Figure 2). No fistulas or abscesses were visualized.

## Differential diagnosis

The leading diagnosis after reviewing the CT scan of abdomen was gastric and colonic erosion secondary to laparoscopic gastric band. However, other differential diagnoses included diverticulitis, gastroenteritis and gastrointestinal perforation.

## Treatment

Bariatric surgery was consulted and patient was taken to the operating room for laparoscopic removal of eroded gastric band, repair of gastric erosion and repair of transverse colon erosion.

## Outcome and follow-up

Approximately 5 months after surgery, patient was seen in clinic and was doing well from a GI standpoint. She had lost 8 pounds since the surgery.

## Discussion

Gastric band erosion is an important late complication with an overall incidence of 1.46% of cases as reported in a recent systematic review.^3^ The connecting tube may also cause erosion of the distal colon. When gastric band erosion is identified on CT scan, it is important to trace the length of the connecting tube for possible involvement of the distal GI tract, as simultaneous erosion of the colon has been documented.^4,5^ In two previously documented cases, gastrocolic fistulas were formed.^4,5^ One of these patients also had an associated abscess formation.^5^ Yet another case reported evidence of small bowel obstruction secondary to connection tube.^6^ Our case is unique in that there was no evidence of associated fistula formation, abscess or small bowel obstruction.

Other rare complications of LAGB include stomal stenosis, malpositioned bands, pouch dilation, band slippage, perforation, gastric volvulus, intraluminal band erosion and port- and band-related issues.^7,8^ Clinical presentation of gastrointestinal erosion may include nausea, emesis, abdominal pain and hematochezia, or patient may be asymptomatic. Of all cases with simultaneous gastric and colonic erosion, our patient was the only one to report haematochezia as the presenting symptom. This may be related to the patient’s long-term use of anticoagulants. Endoscopy and/or colonoscopy may show foreign body as seen in our case. Furthermore, CT scan of the abdomen may show displaced gastric band inferior to gastric fundus, erosion of band into the gastric wall, erosion of connecting tube into colon with surrounding inflammatory changes, gastrocolic fistula or abscess. In previous documented cases, the complete diagnosis of gastric and colonic erosion was confirmed with the addition of gastroscopy or during surgery in addition to CT findings. In contrast, CT scan alone was sufficient to confirm both gastric and colonic erosion in our patient. Treatment involved band removal with repair of gastric and colonic erosions using laparoscopic technique, which was similar to previous cases.

An important limitation of this case report includes obtaining the CT scan with oral contrast only, instead of the correct approach of with and without IV contrast medium. In patients with suspicion of GI bleeding, the use of IV contrast allows the radiologist evaluate for active bleeding and ischemic changes in the bowel walls. It also increases the sensitivity for distinction between the visceral wall and the laparoscopic gastric band device. Unfortunately, this study was ordered and performed with oral contrast only.

Several mechanisms have been suggested to correlate with band erosion, including chronic ischaemia secondary to gastric wall pressure from the band, suturing the band to the stomach, infection and even number of cases performed by the bariatric surgeon.^3,9,10^ Given that band erosion is a late complication that may not appear for several years after the procedure, it is important to have a low threshold for this diagnosis. We hope that this case report will help radiologists and clinicians to keep this rare complication on their differential diagnosis in future cases with similar presentations.

## Learning points

1. In a patient with laparoscopic gastric band presenting with haematochezia, nausea, emesis and abdominal pain, band erosion should be considered in the differential diagnosis.
2. Once gastric band erosion is identified on CT, one should trace the length of the connecting tube in search for possible colonic erosion.
3. Treatment for simultaneous gastric and colonic erosion involves band removal with repair of gastric and colonic erosions using laparoscopic technique.

## Consent

Written informed consent for the case to be published (including images, case history and data) was obtained from the patient(s) for publication of this case report, including accompanying images. 
